# Molecular pathways in vascular cognitive impairment and dementia: focus on synaptic plasticity and epigenetic modifications

**DOI:** 10.3389/fnagi.2026.1741558

**Published:** 2026-01-22

**Authors:** Chuanqiang Liu, Fuyue Li, Luyao Qiao, Baichao Kai, Zuobin Wang, Na Zheng, Shengqiao Wang, Ying Gao

**Affiliations:** 1 First Teaching Hospital of Tianjin University of Traditional Chinese Medicine, Tianjin, China; 2 National Clinical Research Center for Chinese Medicine, Tianjin, China; 3 School of Acupuncture and Moxibustion and Tuina, Tianjin University of Traditional Chinese Medicine, Tianjin, China

**Keywords:** DNA methylation, epigenetic modifications, hippocampal synaptic plasticity, histone modification, neuroimaging, non-coding RNA, vascular cognitive impairment, vascular dementia

## Abstract

**Background:**

Vascular cognitive impairment (VCI) is a group of cognitive disorders caused by cerebrovascular disease and is the second leading cause of dementia. VCI prevalence has significantly increased over the past decade. However, the molecular mechanisms underlying VCI remain unclear.

**Objective:**

This review summarizes recent reports on the critical roles of hippocampal synaptic plasticity and epigenetic changes in VCI and vascular dementia (VaD) by incorporating findings from neuroimaging and molecular biology.

**Methods:**

We reviewed studies employing molecular, electrophysiological, and neuroimaging approaches, conducted over the last two decades. Key targets of investigation included cerebral blood flow regulation, synaptic transmission, and epigenetic mechanisms such as DNA methylation, histone modification, and noncoding RNA regulation.

**Results:**

Growing evidence suggests that chronic cerebral hypoperfusion and microvascular injury cause deficits in hippocampal synaptic plasticity, leading to long-term potentiation and memory formation deficits. Aberrant epigenetic changes, such as dysregulated DNA methylation, histone acetylation, and miRNA expression, contribute to neuroinflammatory and neurodegenerative processes. Electroencephalography and functional magnetic resonance imaging studies reflect changes in neural connectivity and network dynamics, and molecular imaging provides molecular-level evidence of these changes.

**Conclusion:**

VCI is caused by the complex interaction of vascular dysfunction, synaptic dysregulation, and epigenetic modification. Identification of these convergent mechanisms may pave the way for new diagnostic biomarkers and therapeutic targets. Future studies on neuroimaging, molecular profiling, and epigenetic modifications could facilitate the early detection and precision-based treatment of VCI and VaD.

## 1 Background

Vascular cognitive impairment (VCI), caused by cerebrovascular disease, is the most common cause of cognitive impairment after Alzheimer’s disease. It mostly affects people over 65 years of age (Rundek et al., 2022). The World Health Organization estimates that 47 million people worldwide have dementia, and that 30–40% of these cases are associated with cerebrovascular disease (stroke, small vessel disease, etc.) (Smith, 2017). The clinical manifestations of VCI are heterogeneous, ranging from mild deficits in attention, memory, and executive function to severe vascular dementia. In severe cases, patients experience language disturbances, disorientation, and limited daily activities (van der Flier et al., 2018).

The primary pathology of VCI is decreased or interrupted cerebral blood flow, a condition that directly results in insufficient oxygen and nutrient delivery to brain cells (Rajeev et al., 2023). Cerebrovascular diseases, including small vessel disease, white matter lesions, and microvascular damage, disrupt neural networks and impair functional connectivity between brain regions (Wang et al., 2022). As VCI progresses, secondary pathologies such as neuroinflammation and oxidative stress occur and interact synergistically, causing further neuronal damage (Linton et al., 2021). Despite extensive research on VCI, its pathophysiology remains unclear, particularly at the molecular level. Ongoing studies are mainly focused on identifying reliable biomarkers and possible therapeutic targets to improve early diagnosis and treatment (Masserini et al., 2023).

VCI can be classified into several subtypes based on different pathologies and clinical characteristics. Post-stroke cognitive impairment is one of the most common types of VCI that develops after acute stroke (Ip et al., 2024). Cognitive deficits occur in brain regions at or near the stroke site, and affected individuals often experience memory loss, language loss, attention loss, and executive impairment (Cramer et al., 2022). The other frequent subtype is cerebral small vessel disease (CSVD), characterized by chronic pathological changes and long-standing inadequate perfusion (Chen et al., 2018); this subtype presents with white matter lesions, microvascular damage, and deficiencies in executive function, attention, and processing speed (Chen et al., 2017). Mixed cognitive impairment is observed when neurodegeneration and cerebrovascular pathology co-occur; cognitive loss in this condition is driven by both vascular damage and memory loss characteristic of Alzheimer’s disease (Ip et al., 2024).

The most severe type of VCI is vascular dementia (VaD), characterized by cognitive impairment, which seriously affects patient quality of life (Rundek et al., 2022). Patients with VaD present a rapid decline in mental function, decreased ability to perform daily activities, and neuropsychological symptoms such as depression and anxiety (Ng et al., 2025). VaD endangers individual lives and imposes huge emotional and economic burden on families and society. Long-term studies indicate that approximately 60% of patients with vascular dementia are unable to live independently within 5 years of diagnosis (Harrison et al., 2022).

Chronic cerebral hypoperfusion is usually exacerbated by chronic diseases such as diabetes and hypertension. It is a key driver of VCI, resulting in an insufficient oxygen and nutrient supply to the brain tissue, thereby causing neuronal damage (Rajeev et al., 2023). Hypoperfusion directly affects synaptic transmission and neuronal function in important regions such as the white matter and hippocampus. Long-term effects include local ischemia and neurodegenerative processes, such as loss of neuronal function and decreased synaptic plasticity (Jing et al., 2015).

The main pathological changes associated with VCI include microvascular injuries and white matter lesions. Microvascular injury manifests as the thickening, fibrosis, and hardening of small vessel walls, which hinder local perfusion, thereby aggravating cerebral ischemia (Zhang et al., 2019). White matter lesions present as high-signal areas on neuroimaging, are usually associated with microvascular injury, and are involved in progressive cognitive decline (VCI) (Morrison et al., 2022). White matter lesions disrupt cortical and subcortical neural network connections, causing deficits in executive function, attention, and processing speed and are negatively correlated with cognitive function in patients with VCI (Ma et al., 2022).

Along with vascular and structural abnormalities, neuroinflammation and oxidative stress are the most significant pathological changes associated with VCI. Chronic cerebral hypoperfusion prompts local immune responses to activate glial and immune cells and release pro-inflammatory cytokines, causing a series of cascade reactions that damage vascular endothelial cells, affect neuronal function, and lead to neuronal death (Poh et al., 2022). Further, enhanced oxidative stress, which causes reactive oxygen species and free radicals to damage cell membranes, proteins, and DNA, further aggravates neuronal damage (Rajeev et al., 2022).

**Figure 1:**
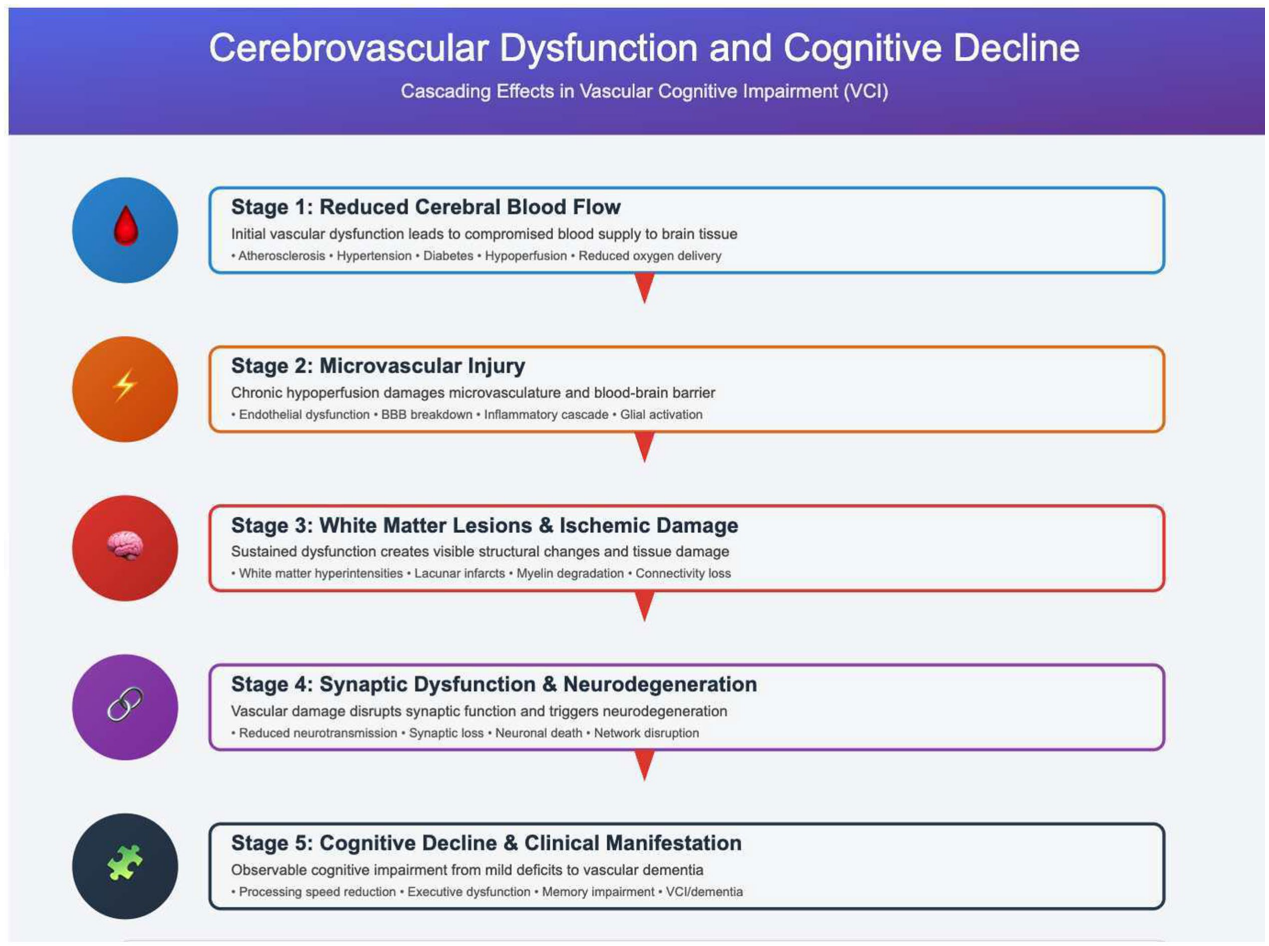
The chronic cerebral hypoperfusion (CCH)-driven multi-cascade pathological model. The core pathogenesis of vascular cognitive impairment (VCI) originates from the reduction or interruption of cerebral blood flow caused by cerebrovascular disease, which leads to ischemia and hypoxia in brain cells. This initial event triggers a series of interrelated secondary pathological processes, with neuroinflammation and oxidative stress serving as key driving factors. Within this process, microvascular injury and white matter lesions are pivotal connecting links. Microvascular dysfunction, an early and persistent microvascular constriction driven by dysregulation of vasoactive neuropeptides such as CGRP1, subsequently leads to the disruption of blood–brain barrier (BBB) integrity. Molecules like Lipocalin 2 (LCN2) mediate this process by promoting endothelial-mesenchymal transition (EndMT) and peripheral neutrophil infiltration. White matter injury is primarily attributed to impaired differentiation of oligodendrocyte precursor cells (OPCs) and compromised remyelination. Ultimately, multiple pathological changes act synergistically to destroy the neural network structure, prominently manifesting as synaptic dysfunction (e.g., glutamatergic synaptic imbalance, downregulation of synaptic proteins) and neuronal apoptosis. This cascade, from reduced cerebral blood flow to synaptic loss, clinically drives the progression from mild cognitive impairment to severe vascular dementia.

Therefore, the pathogenesis of VCI involves a series of pathophysiological changes resulting from the synergistic effects of multiple factors, including cerebral blood flow injury, microvascular injury, white matter lesions, neuroinflammation, and oxidative stress (Rajeev et al., 2022) (see Figure 1). Collectively, these mechanisms form the basis of cognitive impairment in patients with VCI, and provide targets for future treatments. However, the specific pathogenesis of VCI still needs to be clarified at the molecular level for the discovery of new biomarkers and better treatment methods for VCI in the future. Therefore, this review summarizes the critical roles of hippocampal synaptic plasticity and epigenetic changes in VCI and vascular dementia (VaD), and indicates the importance of incorporating findings from neuroimaging and molecular biology.

## 2 The role of synaptic plasticity in vascular cognitive impairment and dementia

### 2.1 Fundamental concepts of synaptic plasticity and its role in cognition

The synapse is the basic unit of information transmission between neurons. Synaptic plasticity refers to the ability to adjust the strength or structure of synaptic connections, which enables neurons to respond to external stimuli and lay the foundation for learning and memory mechanisms. Synaptic plasticity takes two forms, long-term potentiation (LTP) and long-term depression (LTD), which represent the enhancement or weakening of synaptic transmission efficiency, respectively, and play an important role in the functional regulation of the brain. The N-methyl-D-aspartate receptor (NMDA-R)/NR2B mediates LTP, and the Ca^2+^ influx mediated by it initiates a series of downstream signaling pathways, allowing the insertion of *α* -amino-3-hydroxy-5-methyl-4-isoxazolpropionic acid (AMPA) receptors to promote changes in synaptic strength and structure (Massicotte, 2000). Calmodulin-dependent protein kinase II (CaMKII), one of the downstream receptors mediating NMDA-R, can mediate synaptic signal transduction and regulate the stability and plasticity of synaptic scaffolds (Yasuda et al., 2022). LTD is another pathway that reduces synaptic strength to maintain network homeostasis and plasticity (Piochon et al., 2016).

Synaptic plasticity involves proteins such as Synaptophysin (SYN) and Postsynaptic Density Protein 95 (PSD-95), which regulate the synaptic structure and function (Vallejo et al., 2016). Neural cell adhesion molecules (NCAM and L1-CAM) play a role in synapse formation or pruning and are involved in regulating the plasticity of neural networks (Moreland and Poulain, 2022). Brain-derived neurotrophic factor (BDNF), one of the most widely studied molecules, activates the ERK/Akt signaling pathway through TrkB receptors and promotes synaptic protein expression (Numakawa et al., 2018). In addition to the ERK/Akt pathway, the CREB/BDNF pathway is also related to plasticity (Numakawa et al., 2018). Quintiapine is reported to downregulate the expression of matrix metalloproteinase-9 (MMP-9) by activating the CREB/BDNF pathway, thereby promoting synaptic plasticity and improving cognitive function (Morella et al., 2020).

The important mechanisms involved in synaptic plasticity regulation during cognitive impairment include REDOX reactions, epigenetic mechanisms, and inflammatory responses of glial cells (K C and Priyadarshini, 2025). DNA methylation and histone acetylation are epigenetic mechanisms involved in regulating the expression of synapse-related genes, thereby influencing learning and memory (Yu et al., 2011). For instance, the epigenetic factor JADE2 promotes the maintenance of synaptic function and cognitive ability in the hippocampus by activating the cytoskeletal protein Rac1 (Fan et al., 2022). During synaptic plasticity regulation involving the microglia and glia, microglia participate in synaptic pruning by regulating inflammatory factors and the complement system, thus maintaining synaptic plasticity and cognitive function, while glia further expand the complex network of synaptic plasticity regulation (Welberg, 2014). Furthermore, increasing evidence indicates that environmental and behavioral characteristics are also important factors that influence synaptic plasticity (Appelbaum et al., 2022).

In diseases like cerebral ischemia, neurodegenerative diseases, and mental disorders, synaptic plasticity damage is considered an important mechanism leading to neural network dysfunction (K C and Priyadarshini, 2025). In neurodegenerative diseases, such as vascular cognitive impairment (VCI) and dementia, disordered synaptic plasticity manifests as a reduction in the number of synapses, a decline in synaptic strength, and abnormal changes in synaptic morphology, leading to the weakening of neural network connections and loss of information processing capabilities (Kozubski et al., 2021). Reduced synaptic transmission efficiency of the CA3-CA1 circuit in the hippocampus, accompanied by long-term enhancement (LTP) disorders, affects learning and memory abilities (Gui et al., 2023). Abnormal post-translational modifications of proteins and dynamic changes in membrane receptors can also affect synaptic strength. For instance, the absence or mutation of farnesyltransferase (FT) and geranylgeranyltransferase (GGT), which are key enzymes involved in protein prenylation, can cause synaptic dysfunction and cognitive problems (Moon et al., 2021). Further, abnormal synaptic plasticity can affect the synchronous activities of neuronal populations, disrupt the overall coordination of neural networks, and exacerbate the decline in cognitive function (Palop and Mucke, 2016).

Improvement of cognitive function has progressed to some extent by addressing abnormal synaptic plasticity. For instance, repetitive transcranial magnetic stimulation (rTMS) has been proven to enhance cognitive function in animal models of cerebral ischemia through the regulation of chemical synaptic transmission and glutamatergic synaptic plasticity (Hong et al., 2020). Exercise intervention improves spatial memory by regulating REDOX reactions, influencing neurotransmitter transmission, and promoting synaptic density and dendrite growth in hippocampal neurons (Zheng et al., 2019). The generation and integration of new neurons are equally important for synaptic plasticity. Regulating neurogenesis in the hippocampus can improve memory function in Alzheimer’s disease model mice, showing a beneficial effect on cognitive function by promoting healthy neurogenesis and inhibiting abnormal neurogenesis (Whalley, 2016).

**Figure 2:**
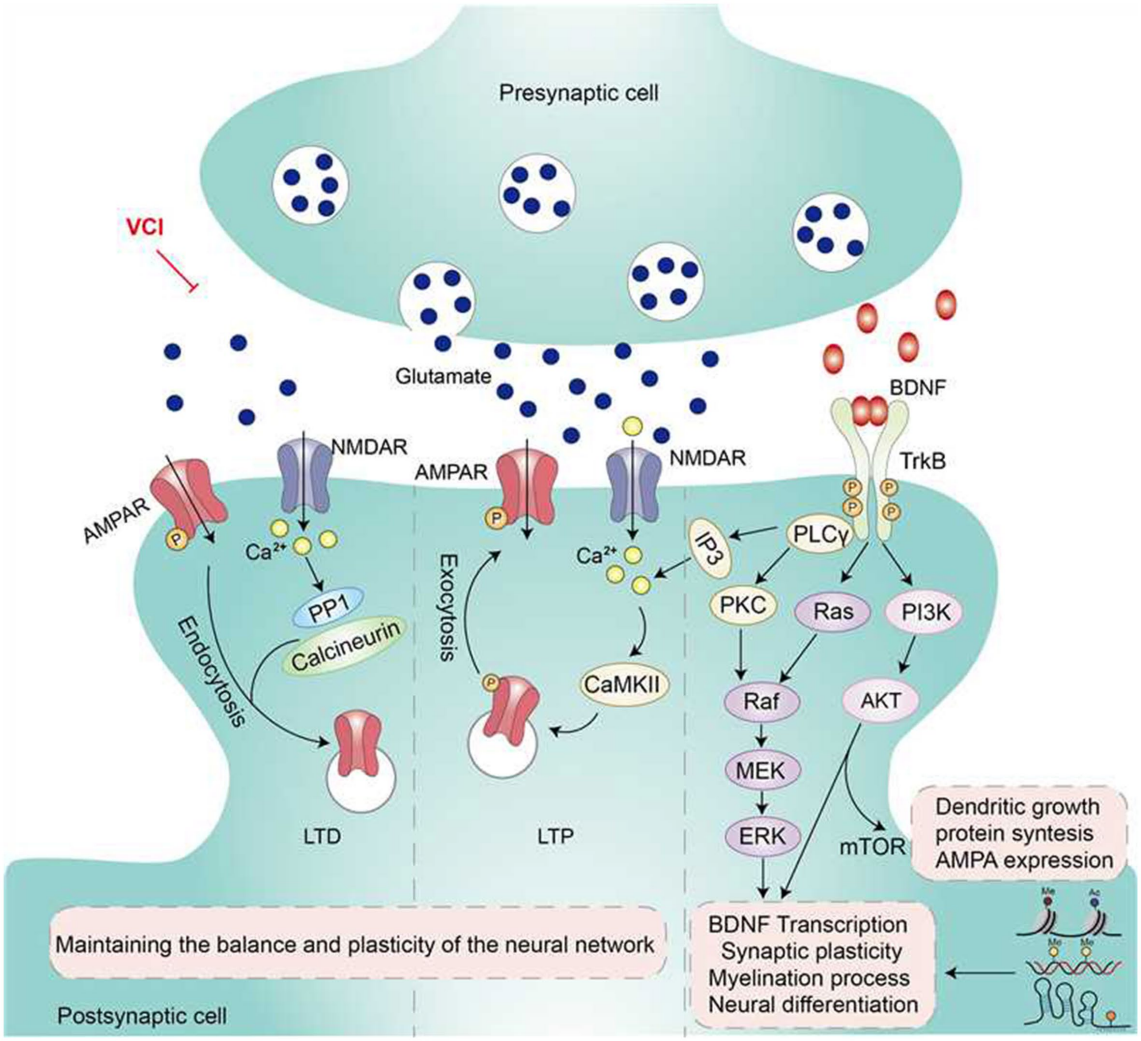
Mechanisms of synaptic plasticity in VCI. Synaptic plasticity is an important foundation for learning and memory. It is mainly influenced by the NMDA/AMPA receptor pathway, important proteins such as CaMKII, and signaling molecules such as BDNF. Pathological factors such as cerebral ischemia can lead to damage or loss of synaptic plasticity, resulting in reduced number of synapses in neurons, weakened synaptic strength, and abnormal changes in synaptic morphology. This affects the functional connections between neurons and causes cognitive dysfunction.

Overall, synaptic plasticity is regulated through a multilevel molecular mechanism that involves dynamic changes in synaptic protein components, interactions between signaling pathways, epigenetic modifications, and the roles of glial cells (Kozubski et al., 2021). Further research on the related mechanisms can better explain the pathogenesis of cognitive impairment as well as provide new molecular targets and theoretical references for research on vascular cognitive impairment, dementia, and other diseases (see Figure 2).

## 3 Epigenetic modifications and their role in vascular cognitive impairment

Epigenetic modifications mainly include DNA methylation, histone modification, and non-coding RNAs (such as miRNAs), which affect neural development and synaptic remodeling by regulating gene expression without DNA sequence alteration. DNA methylation, a key epigenetic modification mechanism, has drawn increasing attention for its role in VCI. The following section focuses on the mechanism of DNA methylation and its regulatory functions, as well as the effects of abnormal patterns of DNA methylation.

### 3.1 Mechanisms and regulatory functions of DNA methylation

DNA methylation is the process of adding -CH3 to the 5-position carbon atom of the cytosine (C) base to form 5-methylcytosine (5mC) in a DNA molecule and is an important step in epigenetic regulation (Nomura and Morita, 2022). Previous studies suggest that this marker, which mainly functions to “silence” the gene transcriptional activity in the CpG island of a gene promoter and thus regulates gene expression levels (Nomura and Morita, 2022). A CpG island is a DNA sequence containing the cytosine-guanine (CG) dinucleotide composition sequence, usually located near the gene promoter (Angeloni and Bogdanovic, 2021). If a CpG island is methylated, the gene cannot be expressed. Conversely, it may activate the transcription of this gene (Angeloni and Bogdanovic, 2021). Methylation of the CpG island, on the one hand, inhibits the binding of transcription factors, and on the other hand, recruit inhibitory complexes such as MBD, thus blocking gene transcription. In addition to the genome and repeat sequences, CpG methylation is involved in various genomic contexts, such as transcription start sites. For instance, methylation in the promoter region generally indicates that the gene is turned off, whereas methylation in the enhancer region may play a role in both promoting or inhibiting gene expression (Zardo, 2021).

DNA methylation is catalyzed by a class of specific enzymes called DNA methyltransferases (DNMTs). In mammals, the main DNMTs are DNMT1, DNMT3a and DNMT3b. DNMT1 is a maintenance methyltransferase responsible for transferring the methylation status of the mother chain to the newly synthesized daughter chain during replication, thereby maintaining the DNA methylation map of the cell. DNMT3a and DNMT3b are *de novo* methyltransferases involved in establishing new genomic methylations (Haggerty et al., 2021). DNMT activity is influenced by multiple regulatory factors, such as post-translational modifications of proteins, regulation of gene expression, and protein–protein interactions (Kaplun et al., 2021). For example, DNMT1 binds to semi-methylated DNA, interacts with histone methylation markers (such as H3K9me2/3), and participates in the maintenance of methylation during replication (Karemaker and Baubec, 2020). In Further, demethylases, such as TET family proteins (TET1/2/3), can oxidize 5mC to 5-hydroxymethylcytosine (5hmC) and promote DNA demethylation, constituting a key link in the dynamic regulation of DNA methylation.

DNA methylation maintains a dynamic equilibrium through methylases and demethylases, thereby making the genomic methylation map both stable and plastic. The Kaiso protein recognizes methylated DNA, and recruits related complexes to regulate local DNA methylation levels, thereby regulating gene expression and cell fate (Kaplun et al., 2021). The external environment, metabolic conditions, and even drug treatment can alter the DNA methylation status by regulating the expression and activity of DNMTs or TETs, thereby influencing gene expression and cell function. Further, melatonin can affect DNA methylation status by regulating the expression of DNMTs and TETs, demonstrating an epigenetic modification regulatory effect (Linowiecka et al., 2023).

In summary, DNA methylation mainly inhibits gene transcription through CpG island methylation and is jointly regulated by methyltransferases and demethylases, including DNMTs and TETs (Zardo, 2021). A regulatory network of mutual checks, balances, and coordination is formed among various methyltransferases and demethylases, which are highly complex and multilevel. Understanding this mechanism can reveal the regulatory mechanism of gene transcription and provide an important theoretical basis for understanding the pathogenesis and development of VCI and dementia.

### 3.2 Abnormal patterns of DNA methylation in VCI

Methylomics analysis revealed an abnormal DNA methylation spectrum in the brain tissues of patients with VCI (including Brodmann region 7). Methylation differences at CpG sites in 19 patients with VCI and 21 controls were compared and analyzed using the Illumina EPIC methylation chip platform. Eventually, significant differential methylation was observed at 3,601 sites, and more than 82% of these sites were hypermethylated (Vishweswaraiah et al., 2025). This indicates a widespread genome-wide hypermethylation phenomenon in the brain tissues of patients with VCI, which may mainly affect genes, such as MTCH2, DPRX, and DENND4A, which are related to neurological function or closely related to blood vessels. This suggests that abnormal methylation can affect the function of neurons and vascular cells, subsequently resulting in the occurrence and development of cognitive impairment (Vishweswaraiah et al., 2025).

Functionally defective DENND4A because of an abnormally methylated gene is an important component in the molecular mechanism of VCI pathogenesis. It encodes the RabGEF protein that regulates Rab proteins and affects vesicle transport and circulation. Excessive methylation can downregulate its expression, resulting in reduced release of synaptic vesicles, decreased signal transmission between neurons, reduced synaptic plasticity, and cognitive decline (Vadla et al., 2023). MTCH2 (Mitochondrial carrier 2) encodes a mitochondrial membrane transport protein that mediates cell metabolism and apoptosis. Hypermethylation and decreased expression of MTCH2 weakens mitochondrial function, reduces the energy supply to neurons, leads to vascular endothelial cell dysfunction, reduces cerebral blood perfusion, and aggravates cognitive impairment. The DPRX (Divergent paired-related homeobox) gene is involved in neurogenesis, and its abnormal methylation affect the developmental survival of neurons (Peng et al., 2024).

Furthermore, gene-set enrichment analysis (GSEA) has revealed that differentially methylated genes are highly enriched in neuronal development, synaptic plasticity, and vessel regulation. These results are consistent with the known pathology of VCI and suggest that epigenetic dysregulation contributes to white matter abnormalities that are characteristic of the disease. Methylation abnormalities show sex-based differences. Male patients are more likely to show abnormal methylation enrichment in vascular regulatory pathways, whereas female patients are more likely to show abnormal methylation enrichment in pathways related to synaptic plasticity and neural development. Therefore, different sexes may need different treatment strategies (Vishweswaraiah et al., 2025). Further, differentially methylated regions (DMRs) around the RAB12 gene suggest that lysosomal dysfunction is a factor that impairs both cognitive and vascular functions, providing a more comprehensive understanding of the molecular mechanism of VCI (Zhao et al., 2025).

**Figure 3:**
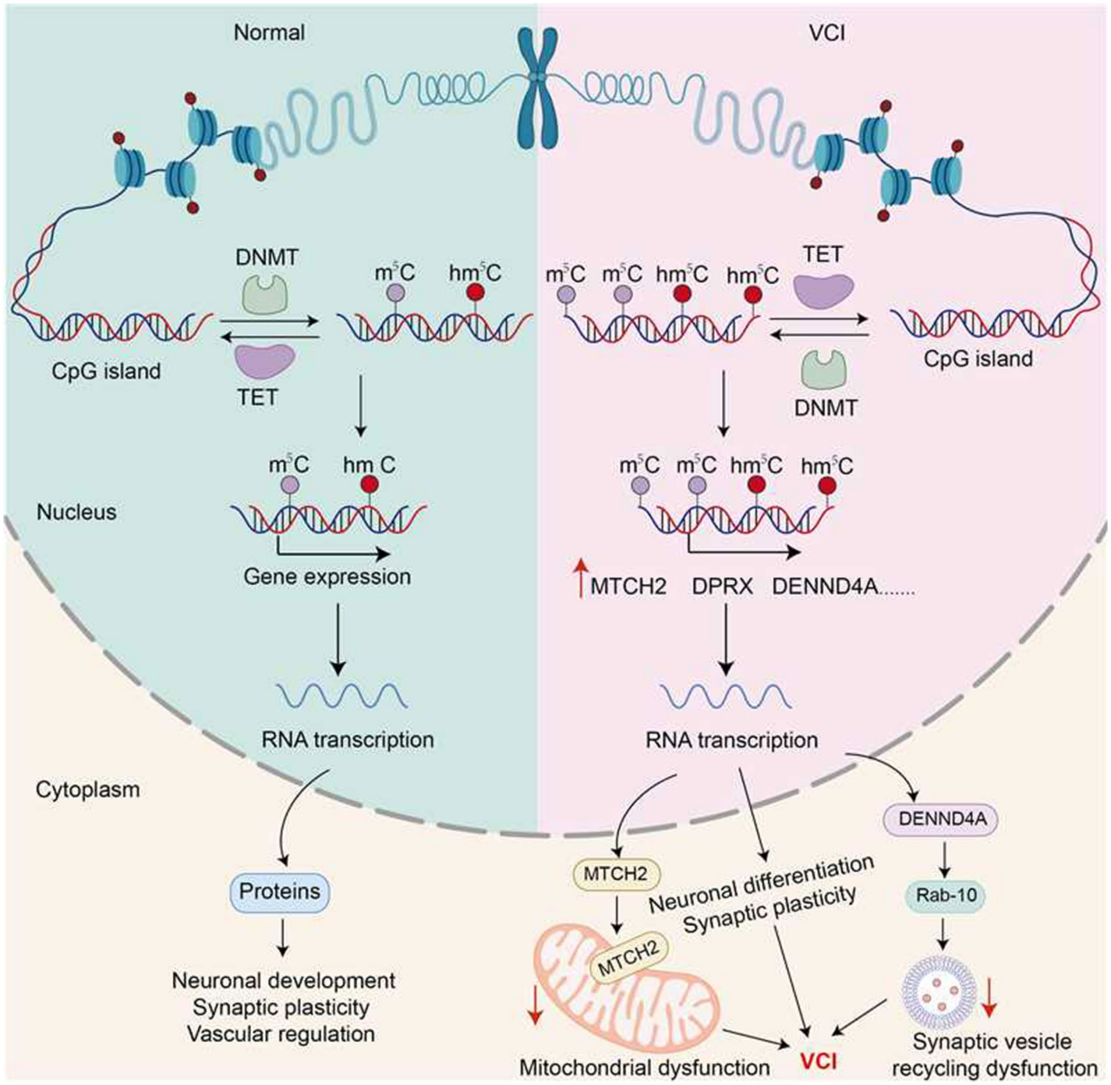
Epigenetic mechanisms in VCI. DNA methylation is a dynamically balanced mechanism involving methyltransferases and demethylases that regulate gene expression. In the brain tissues of patients with VCI, many genomic hypermethylation sites are found, and most of these abnormally methylated sites are located in pathways related to neuronal development, synaptic plasticity, and vascular regulation. This led inhibited the expression of key genes (such as *DENND4A* and *MTCH2*) in the corresponding pathways. Such epigenetic disorders can damage neurons and further lead to synaptic dysfunction, mitochondrial energy supply disorders, and vascular dysfunction, thereby accelerating the occurrence and development of cognitive impairment in a coordinated manner.

In summary, the brain tissues of patients with VCI has an abnormal DNA methylation pattern characterized by hypermethylation. Abnormal methylation patterns can lead to abnormalities in important genes, such as DENND4A and MTCH2, resulting in synaptic dysfunction, mitochondrial metabolic disorders, and vascular damage, thereby triggering disease progression. Moreover, methylation abnormalities exhibit sex- and pathway-specific characteristics (see Figure 3). Overall, obvious epigenetic change patterns are observed in VCI, which is conducive to pre-disease prevention, early diagnosis, and individualized treatment. Simultaneously, this finding suggests that combined neuronal and vascular co-treatment should be emphasized.

## 4 Applications of neuroimaging techniques in VCI research

Electroencephalography (EEG) utilizes brain electrical activity to evaluate neurological function and has been applied in the study of neuropathology. In VCI research, EEG is used to explore the changes in neural electrical activity and its impact on synaptic plasticity and neural networks (de Almeida et al., 2021). Moreover, EEG can record electrical signals between various brain regions in real time, thereby revealing the neurophysiological processes of VCI. Compared with the healthy control group, the EEG data of patients with VCI showed differences in the electrical activity patterns of different brain regions related to cognition, executive function, and memory. Patients with VCI show reduced high-frequency (*α* waves and *β* waves) activity and increased low-frequency (*θ* waves and *δ* waves) activity. Both these changes are associated with cognitive impairment (Ma et al., 2022). Chronic cerebral hypoperfusion leads to high low-frequency activity and low high-frequency activity in the hippocampus (Zhang et al., 2025). By analyzing the frequency bands related to synaptic plasticity (i.e., *α* waves and *β* waves), valuable information about the functional connectivity and synaptic connection changes of the brain in VCI can be obtained.

Further, electroencephalography (EEG) can reflect the real-time dynamic changes in neural networks and is used to detect and locate regional brain dysfunction in patients with VCI (Gavaret et al., 2023). Patients with VCI often present with abnormal characteristics such as loss of phase and amplitude synchrony in the brain regions, leading to network dysfunction and changes in synaptic plasticity. Therefore, EEG is an important tool for explaining disease mechanisms and offers the potential for early disease diagnosis and treatment.

### 4.1 Linking functional magnetic resonance imaging (fMRI) to molecular mechanisms

Functional magnetic resonance imaging (fMRI) uses changes in blood oxygen level-dependent (BOLD) signals as markers of neuronal activity to measure brain activity; this signal is indirectly associated with neuronal activation (Mervin et al., 2020). Most fMRI studies on VCI use BOLD technology to explore the functional connectivity of brain regions or synaptic plasticity-related variations among patients (Chen et al., 2017). Compared with EEG, which provides high temporal resolution but limited spatial resolution, fMRI can depict the network disorders in patients with VCI more accurately.

The results of multiple fMRI studies have shown that functional regions such as the hippocampus, prefrontal cortex, and parietal lobe in patients with VCI have low activity or dysfunction (Zhang et al., 2025). Cerebrovascular diseases (decreased CBF and small vessel disease) disrupt the functional connections between these functional areas, and tasks involving advanced cognitive functions (such as processing, language, and memory) are found to be associated with decreased inter-regional communication based on fMRI visualization (Wang et al., 2025). Additionally, fMRI can describe synaptic plasticity and network dynamics in chronic ischemic conditions caused by cerebrovascular diseases. In this case, the reactivity of neurons and synaptic efficacy both decline, leading to cognitive impairment in the human body (Ahmed-Popova et al., 2020).

Based on the above studies, the combination of functional magnetic resonance imaging (fMRI) with molecular biology to explore the molecular basis of VCI in clinical practice is a new approach (Zhang et al., 2019). For instance, by using gene expression profiles to correlate fMRI scans, changes in brain function under different pathological conditions can be matched with gene expression patterns in pathways such as neuroinflammation and neuroprotection (Wei et al., 2023).

### 4.2 Integration of molecular imaging and gene expression analysis

Molecular imaging is an emerging imaging method combining traditional imaging techniques with molecular biology techniques to image specific molecules in real time. It can be used to measure molecular changes in living organisms and provides highly detailed insights into diseases. In neuroscience, particularly VCI, molecular imaging has become increasingly important for studying the molecular basis of brain diseases (Villemagne and Li, 2022).

In VCI, molecular imaging is used to describe cerebral blood flow impairment, vascular disease, and brain damage, as well as for real-time monitoring of the expression and distribution of critical molecules, such as inflammatory cytokines, neurotrophic factors, and angiogenic proteins. Together with gene expression studies, molecular imaging can help understand how epigenetic changes, such as DNA methylation and histone alterations, affect brain function (Wei et al., 2023). By labeling specific receptors, proteins, or nucleic acids, molecular imaging can provide information on the spatial and temporal changes associated with VCI pathology. When compared with gene expression data, this information can help explain epigenetic regulation (Lau et al., 2023).

**Figure 4:**
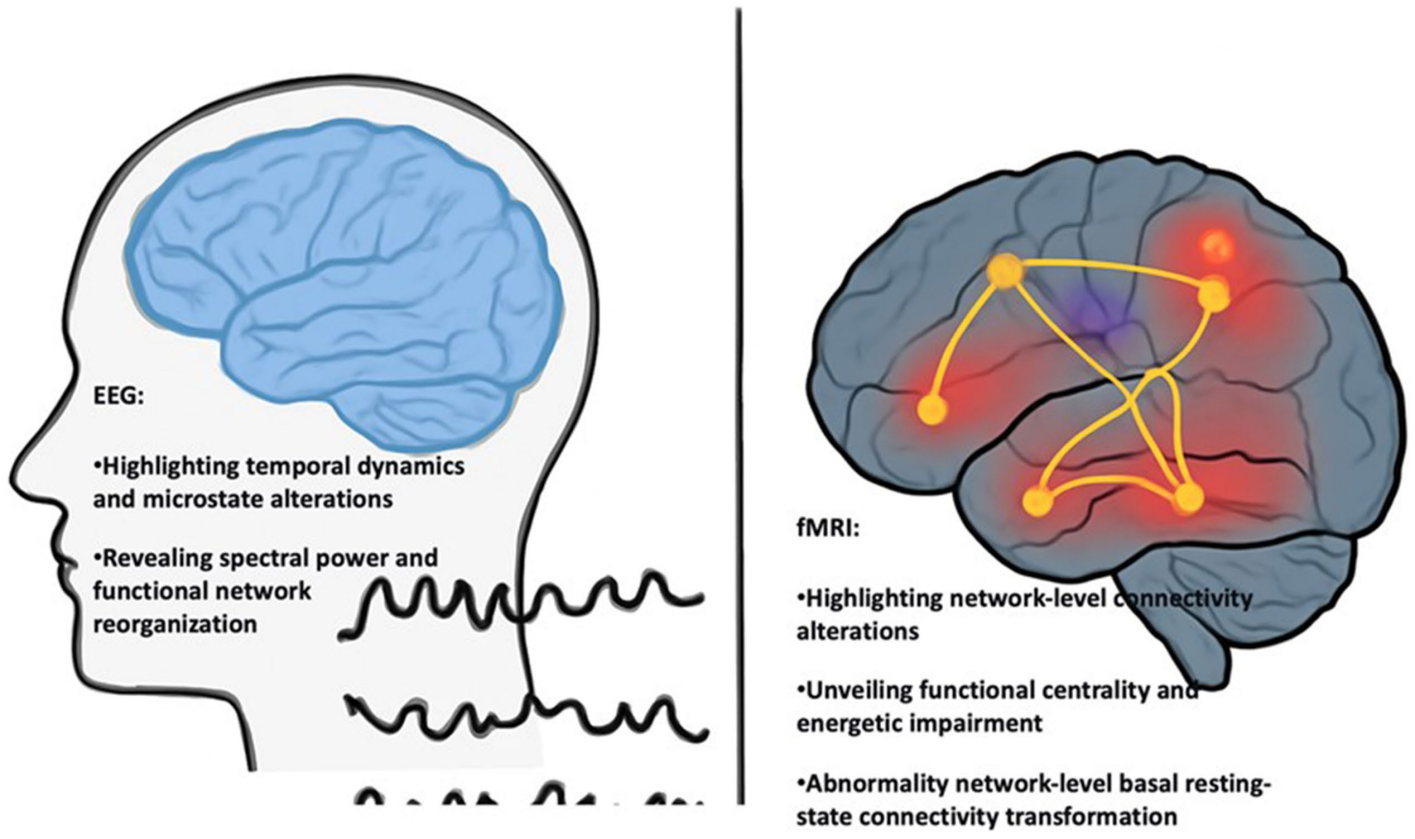
Comparison of EEG and fMRI in VCI research. EEG and fMRI provide complementary information about brain function in VCI. EEG emphasizes neural electrical activity and reveals the temporal dynamics, microstate changes, and power and functional network reorganization; fMRI evaluates network connectivity changes, functional centrality and energetic impairments, and abnormal basal resting state connectivity changes.

In combination with gene expression studies, molecular imaging enables the detection of neuroinflammatory molecules in patients with VCI. This integrated approach can explain how these molecules influence gene transcription through epigenetic processes that regulate neuroinflammatory responses. Moreover, molecular imaging can provide insights into how brain health is linked to gene regulation and how epigenetic processes regulate angiogenesis and vasomotor activity, which affect cerebral blood flow and cognitive performance (Hanania et al., 2024). For example, dysregulation of vasoactive neuropeptides (such as CGRP) is known to be a key driver of microvascular constriction in chronic cerebral hypoperfusion (CCH) and microvascular dysfunction precedes and drives classical neuropathological changes (Császár et al., 2022). The combination of molecular imaging and molecular biology techniques allows researchers to obtain a multidimensional view of VCI molecular processes. In addition to improving our understanding of the disease pathogenesis and evolution, integration of molecular methods can lead to the development of new diagnostic biomarkers and personalized treatment approaches for VCI (see Figure 4).

## 5 Challenges and future perspectives in VCI research

The molecular basis of VCI and its associated dementia, particularly synaptic plasticity and epigenetic changes, is limited by the lack of human postmortem tissue. For example, a recent epigenomic study including19 patients with VCI and 21 controls, focused exclusively on Brodmann area 7 (Hainsworth, 2022). Such a small sample size limits the statistical power and generalizability of the results and makes it difficult to capture all the complex heterogeneous molecular phenotypes of VCI. Although this region is involved in cognition, focusing on a single brain region is a caveat. VCI is a diffuse disorder that affects cortical, subcortical, and white matter tracts. The epigenetic landscape and its functional consequences are likely region-specific. Therefore, single-region studies provide only partial and potentially misleading insights (Xu et al., 2021).

The second, and more fundamental, challenge is causality. Although studies have consistently demonstrated a strong link between aberrant DNA methylation patterns and VCI, the direction of causality remains unclear. Are epigenetic changes driving pathogenesis, the downstream consequences of vascular injury and neurodegeneration, or both? Disentangling the causes from the effects in human studies is difficult. Finally, a key challenge in VCI research is the integration of multimodal neuroimaging with molecular biology. EEG and fMRI provide valuable macroscale data on brain activity and connectivity, but do lack the resolution to probe molecular mechanisms. In contrast, molecular data provide details but lack spatial and functional contexts. A synergistic approach combining imaging phenotypes and molecular data (e.g., transcriptomics and epigenomics) can bridge this gap and allow us to better understand VCI as well as identify novel diagnostic biomarkers and therapeutic targets (Vishweswaraiah et al., 2025).

### 5.1 Therapeutic potential

With a deeper understanding of the molecular basis of VCI, therapeutic strategies beyond current approaches emerge. Future treatment directions include using epigenetic biomarkers for early diagnosis, implementing combined interventions targeting synaptic plasticity and vascular health, and advancing the clinical translation of sex-specific therapies (Bordet et al., 2017). Multiple therapeutic strategies are required to address synaptic dysfunction and cerebrovascular impairment. Several promising candidates have been identified in preclinical studies (Ibrahim and Gabr, 2019).

For example, supplementation with exogenous branched-chain amino acids (BCAAs) can ameliorate cognitive impairment by regulating glutamate metabolism and transport in rats with chronic cerebral hypoperfusion, thereby improving synaptic structure and function (Ragni et al., 2023). Resveratrol exerts neuroprotective effects in a CCH rat model by inhibiting hippocampal apoptosis and enhancing synaptic plasticity (Sarroca et al., 2021). Salidroside attenuates neuroinflammation and neuronal apoptosis by modulating microglial transition from a pro-inflammatory M1 phenotype to an anti-inflammatory M2 phenotype (Fan et al., 2021). Hyperbaric oxygen therapy (HBOT) confers robust neuroprotection against CCH-induced VCI by upregulating miR-137-3p and suppressing its target TRAF3, thereby inhibiting the TAK1/NF-κB pathway and reducing neuroinflammation and apoptosis (Bin-Alamer et al., 2024). Further, compounds targeting oligodendrocyte precursor cells (OPCs), including beraprost sodium and 13-docosenamide, promote remyelination by facilitating OPC proliferation and differentiation, alleviating white matter injury, and improving cognitive function (Shao et al., 2021). Combining multiple approaches to simultaneously restore synaptic plasticity and protect cerebrovascular function thus holds promise for achieving comprehensive therapy (Lyden, 2021).

## 6 Conclusion

The pathogenesis of VCI/VD is multifaceted and involves a series of interactive processes that are jointly mediated by abnormal neuronal function and vascular lesions. Abnormalities in synaptic plasticity and epigenetic modifications (such as DNA methylation), play significant roles in the pathogenesis of VCI. In particular, highly methylated DNA can stably and precisely regulate the expression of genes related to neurovascular systems, revealing the molecular basis of VCI pathogenesis and providing new ideas for the target exploration, diagnosis, and treatment of VCI in future.

Although current research has made certain achievements, many questions regarding the molecular mechanism of VCI remain unsolved. In future studies, new technologies such as single-cell sequencing and spatial transcriptomics should be applied to further analyze the epigenetic regulatory network of VCI in different brain regions or cell types. Meanwhile, long-term longitudinal cohort studies can further reveal the relationship between changes in DNA methylation levels and disease progression, and thus, can be used as an early warning regarding the disease.

In future, the diagnosis, treatment, and research on VCI is expected to increasingly feature multidisciplinary intersections. A deeper understanding of the VCI can be achieved by integrating knowledge from the fields of neuroscience, molecular biology, clinical medicine, and neuroimaging. The process of transforming molecular knowledge into clinical applications will be accelerated, and more information related to molecular biomarkers will be obtained. Neuroscientists and clinicians can work together, and new treatment methods can be rapidly scaled from the laboratory to clinical practice. Collaborations between molecular biologists and imaging technicians can help provide a deeper understanding of how molecular markers reflect neural functional states and other issues. Driven by cross-border cooperation, the diagnosis, treatment, and prevention of VCI is expected to undergo significant changes in the future.

In summary, related research on VCI is at a stage of promising prospects. As we gain a deeper understanding regarding molecular mechanisms including epigenetic modifications such as DNA methylation, and on this basis, by integrating neuroimaging and the concept of individualized medicine, early diagnosis and effective intervention measures may be possible in future. This also brings new hope for the diagnosis and treatment of neurodegenerative diseases in the future, gives more patients greater confidence and reduces burden on their families.
